# The risk factors of plasma transfusions during resuscitation in pediatric burn patients: a retrospective study from 2010 to 2021

**DOI:** 10.3389/fped.2025.1608479

**Published:** 2025-11-13

**Authors:** Liqiang Zhu, Xiaorong Zhang, Xingcun Wang, Haiwei Zhang, Pengju Chen, Guangfeng Zhang, Peigang Gao

**Affiliations:** 1 Department of Quality Management, The Central Theatre Air Force Hospital of the Chinese People’s Liberation Army, Datong, Shanxi, China; 2 Department of Burn, The Central Theatre Air Force Hospital of the Chinese People’s Liberation Army, Datong, Shanxi, China

**Keywords:** plasma transfusion, pediatric burn, burn resuscitation, risk factors, pre-hospital remedies

## Abstract

**Objectives:**

To analyze the prevalence and risk factors of plasma transfusions during resuscitation after burns in children.

**Methods:**

A retrospective study was carried out at the Burn Treatment Center of the Central Theatre Air Force Hospital of the People's Liberation Army in Datong City, China. The study included pediatric patients (between the ages of two months and 14 years) who were admitted for burns between January 2010 and December 2021. Details of plasma transfusions during the burn resuscitation process for each child were recorded. The association between risk factors and plasma transfusions was evaluated using non-conditional logistic regression.

**Results:**

Of the children who suffered from burns, 12.89% (*n* = 174/1,350) received plasma transfusions during burn resuscitation. The median amount of plasma transfusions administered was 2.5 units (interquartile range, 1.4). Among the pediatric burn patients who received plasma transfusion, 67.24% did not receive any treatments before being admitted to the hospital, while 14.94% were admitted 6 h or more after the burns occurred. The total body surface area (TBSA) of pediatric burn patients who received plasma transfusion ranged from 10% to 55%, with a median burn area of 20% of the TBSA and a mean burn area of 22% of the TBSA. Non-conditional logistic regression analysis identified TBSA, white blood cell count (WBC), heart rate, and pre-hospital remedies as significant predictors of plasma transfusion.

**Conclusion:**

TBSA (total body surface area), WBC (white blood cell count), heart rate, and pre-hospital interventions can serve as reliable predictors for determining the necessity of plasma transfusions during burn resuscitation. Utilizing these factors can assist blood banks and clinicians in preparing and administering targeted plasma treatments, as well as potentially reducing the overall need for plasma transfusions in pediatric burn patients.

## 1 Introduction

Plasma transfusions are commonly used as a treatment for severely burned patients due to the extensive blood loss that often accompanies burns (1). As an ideal colloid solution for shock resuscitation fluid, plasma plays an effective role in reducing overall volume requirements during burn resuscitation by maintaining intravascular colloid oncotic pressure. Plasma contains various beneficial proteins, such as immunoglobulins, fibronectin, and coagulation factors, which may offer advantages over human albumin solutions. However, plasma transfusions may also lead to severe complications, such as infectious viruses and transfusion-related acute lung injuries, which may result in poorer clinical outcomes (2, 3).

Previous studies focused more on plasma transfusions and its associated risk factors in burn patients, while there is few research on the extent of plasma utilization in pediatric burns (4). It is important to consider the unique physiology of children before administering blood or blood products (5). Burn physicians advocate for limiting the use of blood in clinical treatments based on the potential risks and benefits of plasma transfusions (6, 7). To effectively limit plasma transfusions in pediatric burn patients, it is essential to have a comprehensive understanding of blood transfusions. Given the scarcity of literature on plasma utilization in pediatric burn patients, our objective was to examine plasma transfusions in pediatric burn patients at a major burn center in Datong City, China. This study aims to enhance the clinical appropriateness of plasma utilization during burn resuscitation.

## 2 Methods

This retrospective study was conducted at the burn center of The Central Theatre Air Force Hospital of the Chinese People's Liberation Army. The burn center has 70 beds and is primarily responsible for treating burn patients from Datong and other areas in North China. From January 2010 to December 2021, a total of 1,489 pediatric burn patients, ranging in age from two months to 14 years, were admitted to the center. Patients with insufficient information and those admitted to the burn center more than 48 h after their burns were excluded from the study. Ultimately, 1,350 patients were included in the analysis, which examined patient demographics, time from scald occurrence to hospital admission, pre-hospital remedies, burn etiology, burn extent, vital signs, laboratory test results, plasma transfusions and outcomes. Clinical judgment was used by attending physicians and directors to determine the administration of plasma transfusions during burn resuscitation. In this study, one unit of plasma was equivalent to 200 mL. The study received approval from the medical ethical board of the Central Theatre Air Force Hospital of the PLA under protocol 2022/15.

The software SPSS 25.0 (IBM statistics) was used for statistical analysis. All variables with or without plasma transfusion were compared using the independent-sample t test or the Mann–Whitney U test. Non-conditional logistic regression analyses with backward stepwise regression were performed to identify risk factors associated with plasma transfusions. A *p*-value of less than 0.05 was considered statistically significant.

## 3 Results

Of all 1,350 patients, 174 (12.89%) received plasma transfusion during the burn resuscitation. The majority of the patients (88.51%) underwent multiple plasma transfusion. The median number of plasma transfusion per patient was 3 (IQR, 3), and the median amount of plasma utilization was 2.5 units (IQR, 1.4). The median plasma transfusion per %TBSA was 0.1 unit. Median length of hospital stay (LOS) was 22 days (IQR,21), with a median of 4 days (IQR, 3) in intensive care unit (ICU).

The mean and median ages of pediatric burns with plasma transfusion were 2.49 ± 2.37 years and 1.74(IQR, 1.52) years, respectively. A total of 26 children (14.94%) were <1 year old, 108 children (62.07%) were 1–2 years old, 26 children (14.94%) were 3–5 years old, and 14 children (8.05%) were ≥6 years old. A total of 174 children received plasma transfusion, with 107 boys (61.49%) and 67 girls (38.51%) (male to female ratio of 1.60:1).

A total of 174 pediatric burns with plasma transfusion, 117 children (67.24%) did not receive any treatment after burns, while 57 children (32.76%) received treatment after burns. Among the 57 children who received treatment after burns at home, 12 (21.05%) children received first aid after burns. 9 of the 12 children received cool water treatment. Nevertheless, none of the cooling treatments were sustained for >20 min. The other three children received informal treatment of applying toothpaste,bandaged by family members and smeared with “moist burn ointment”, respectively. 54 children received formal treatments at local medical institutions, intravenous fluids were the most commonly used management method (30, 55.56%), followed by wound dressing (27, 50%), and 19 (35.19%) received wound smearing medication.

The median time for children from getting burned to being admitted to hospital was 3 h (IQR, 2 hours). 32 (18.39%) children were admitted to the hospital within 2 h of being burned, and 116 (66.67%), 18 (10.34%), and 8 children (4.60%) were admitted 2–5 h, 6–11 h, and 12 h or more after burn, respectively.

It was found that 57 (32.76%) pediatric burn patients with plasma transfusion received pre-hospital treatments. It was significantly statistical difference in plasma transfusion between pediatric patients with and without pre-hospital treatments (*χ*^2^ = 23.955, *p* < 0.001). Out of the children who received plasma transfusion, 86.21% (150 children) had burns on multiple body parts, while 13.79% (24 children) had burns on a single body part. The upper limbs were the most common burn site, accounting for 51.78% of cases. The proportion of burn in the head/neck, upper extremity, trunk, lower extremity with plasma transfusion was significantly greater than burns without plasma transfusion (*p* < 0.05).

Scalding was the most common cause of burn from plasma transfusion, accounting for 87.93% of cases, followed by flame burns (10.92%), electrical burns and hot solid burns (1.15%). It was a significant difference in the types of burns between those with and without plasma transfusion (*χ*^2^ = 39.544, *p* < 0.001). Among pediatric burn patients who received plasma transfusion, the affected skin area ranged from 10%–55% of the TBSA. The median scald area was 20% of the TBSA, with a mean scald area of 22% of the TBSA. Additionally, 20.69% of children who received plasma transfusion had a burned area greater than 30%, compared to only 0.09% of those without plasma transfusion. The area of burn for patients with plasma transfusion was significantly larger than those without plasma transfusion (U = 5,531.500, *p* < 0.001). Furthermore, 23.56% of patients with plasma transfusion experienced full-thickness burns. The burns in patients who received plasma transfusion were also deeper compared to those without plasma transfusion (*χ*^2^ = 29.138, *p* < 0.001) (Table 1).

**Table 1:** Demographics and characteristics of pediatric burn patients with and without plasma transfusion, 2010–2021.

Variable	Total (*n* = 1,350)	Plasma transfusion (*n* = 174)	No transfusion (*n* = 1,176)	*p* value
Age, median (IQR)	1.68 (1.43)	1.74 (1.52)	1.67 (1.42)	0.761
Sex, *n* (%)				0.997
Male	830 (61.48)	107 (61.49)	723 (61.48)	
Female	520 (38.52)	67 (38.51)	453 (38.52)	
Address, *n* (%)^a^				0.221
Rural area	976 (82.09)	132 (78.57)	844 (75.16)	
Urban	313 (17.91)	34 (21.43)	279 (24.84)	
Time from scald occurrence to hospital admission (h), median (IQR)	3 (3)	3 (2)	3 (4)	0.140
Pre-hospital remedies, *n* (%)				<0.001
No	674 (49.93)	117 (67.24)	557 (47.36)	
Yes	676 (50.07)	57 (32.76)	619 (52.64)	
External cause of burn, *n* (%)				<0.001
Flame	49 (3.63)	19 (10.92)	30 (2.55)	
Scald	1,209 (89.56)	153 (87.93)	1,056 (89.79)	
Electrical	8 (0.59)	1 (0.57)	7 (0.6)	
Solid	84 (6.22)	1 (0.57)	83 (7.06)	
Degree of burn, *n* (%)				<0.001
Dermal	1,194 (88.44)	133 (76.44)	1,061 (90.22)	
Dermal/full thickness	123 (9.11)	34 (19.54)	89 (7.57)	
Full thickness	33 (2.45)	7 (4.02)	26 (2.21)	
%TBSA, *n* (%)				<0.001
<10%	812 (60.15)	0 (0)	812 (69.05)	
10%∼	420 (37.11)	67 (38.51)	353 (30.02)	
20%∼	81 (6.00)	71 (40.80)	10 (0.85)	
30%∼	37 (2.74)	36 (20.69)	1 (0.09)	
Body location burned, *n* (%)				
Head/neck	503 (37.25)	80 (45.98)	423 (35.97)	0.011
Upper extremity	699 (51.78)	107 (61.49)	592 (50.34)	0.006
Trunk	520 (38.52)	81 (46.55)	439 (37.33)	0.020
Lower extremity	607 (44.96)	103 (59.2)	504 (42.86)	<0.001
Inhalational injury, *n* (%)	8 (0.59)	8 (4.60)	0 (0)	<0.001
Vital signs, mean ± SD				
Temperature (℃)	36.88 ± 0.62	37.02 ± 0.64	36.86 ± 0.62	0.002
Heart rate (bpm)	112.61 ± 23.76	140.83 ± 21.95	108.43 ± 21.02	<0.001
Respiration rate (bpm)	26.75 ± 6.46	32.16 ± 5.90	25.95 ± 6.15	<0.001
Laboratory data, mean ± SD				
White blood cell count (10^9 ^/L)	13.65 ± 5.93	21.19 ± 6.67	12.53 ± 4.92	<0.001
Hemoglobin (g/L)	127.56 ± 18.05	139.17 ± 17.68	125.84 ± 17.47	<0.001
Red blood cell (1,012 /L)	4.67 ± 0.64	5.09 ± 0.62	4.60 ± 0.61	<0.001
Hematocrit (%)	37.02 ± 5.84	38.43 ± 5.64	36.81 ± 5.85	0.001
RDW-CV (%)	14.20 ± 1.67	14.14 ± 1.60	14.21 ± 1.68	0.626
RDW-SD (fL)	41.78 ± 4.37	40.50 ± 3.79	41.97 ± 4.42	<0.001
Platelet counts (10^9 ^/L)	294.35 ± 91.79	328.28 ± 110.84	289.33 ± 87.57	<0.001
Length of stay (days), median (IQR)	11 (10)	22 (21)	10 (8)	<0.001
Hospital costs (RMB), median (IQR)	3,143 (3,934)	8,009 (8,716)	2,803 (2,893)	<0.001

a Missing values address 61.

There was a significant difference in the respiration rate of burn patients based on plasma transfusion (*t* = 12.511, *p* < 0.001). This study revealed that burn children who received plasma transfusion had higher body temperatures (*t* = 3.095, *p* = 0.002). Likewise, individuals who received plasma transfusion had higher heart rates compared to those who did not (*t* = 18.871, *p* < 0.001) (Table 1).

Compared to burn patients without plasma transfusion, those with plasma transfusion had significantly higher white blood cell counts, hemoglobin, red blood cells, hematocrit and platelet counts (*p* < 0.001). While red cell distribution width (RDW-SD) in the burn children with plasma transfusion was lower than those without plasma transfusion (*p* < 0.001). Furthermore, the burn children who received plasma transfusion had significantly longer hospital stays (U = 39,879.000, *p* < 0.001) and higher hospital costs (U = 26,391.000, *p* < 0.001) than those who did not (Table 1).

These variables significantly predicted the need of pediatric burn patients for plasma transfusion include TBSA [OR = 1.516, 95% CI = (1.406; 1.636), *p* < 0.001], WBC [OR = 1.111, 95% CI = (1.056; 1.169), *p* < 0.001], heart rate [OR = 1.023, 95% CI = (1.010; 1.037), *p* < 0.001] and pre-hospital remedies [OR = 0.541, 95% CI = (0.296; 0.986), *p* = 0.045] (Table 2).

**Table 2:** Non-conditional logistic regression model for prediction of plasma transfusion.

Variable	Coefficient	Standard error	*p* value	Odds ratio	95% CI for odds ratio	
					Lower	Upper
TBSA	0.416	0.039	<0.001	1.516	1.406	1.636
White blood cell count	0.105	0.026	<0.001	1.111	1.056	1.169
Heart rate	0.023	0.007	<0.001	1.023	1.010	1.037
Pre-hospital remedies	−0.615	0.307	0.045	0.541	0.296	0.986

## 4 Discussion

Plasma transfusion is a crucial treatment modality for resuscitating patients with major burns. It effectively expands volume and may confer beneficial effects on the endothelium by reducing microvascular leakage that occurs following severe burn injury (8). However, the epidemiology of plasma transfusion in pediatric burn patients in China has not yet been extensively studied (9, 10). To optimize the treatment of burn-injured children, it is important to carefully consider the impact of transfusion on patient outcomes.

In a study conducted by Lu et al. (11), it was found that 44.90% of 89 patients with burns covering 15%–65% of the TBSA received plasma transfusion. Another study, which included 1,615 burn patients, found that 38 individuals (2.35%) received fresh frozen plasma (FFP) transfusion (8), and Jin Jian et al. (12) reported a plasma transfusion rate of 37.17% among 600 patients with an average burn surface area of 45.2% TBSA from 2009 to 2019. In the present study, the overall plasma transfusion rate was 12.89%. Among patients with 10%–19% TBSA burn, the rate was 15.95%. For patients with 20%–29% TBSA burn, the rate was 87.65%. Furthermore, 97.30% of children with a TBSA burn of ≥30% received plasma transfusion, while no transfusion was given for a TBSA of <10%. In Jin Jian et al.'s (12) study, burn patients received an average of 11.4 units of plasma transfusion. In contrast, the median number of units of plasma transfused per patient was 2.5, ranging from 0.5 to 12 units in this study. Which is significantly lower compared to previous studies.

In the shock stage of burn injuries, there is a significant oozing of plasma from the capillaries onto the wound surface and tissue space. To address this issue, plasma is frequently used as an effective colloid fluid for resuscitation to increase the circulating blood volume and prevent excessive fluid accumulation, edema, and pulmonary complications (13). In addition, the administration of plasma has been linked to improving immunity, enhancing nutrition, and increasing the blood albumin levels (14).

The logistic regression analysis of this study revealed that TBSA, WBC, heart rate, and pre-hospital remedies remained statistically significant in predicting plasma transfusion requirements in burned children. Notably, burn area emerged as the most crucial predictive factor for plasma transfusion during burn resuscitation. Non-transfused children had a median TBSA of 7%, while their transfused counterparts had a median TBSA of 20%. Nonetheless, only a few studies were available to explore the association between TBSA and plasma transfusion in pediatric burn patients. One such study conducted by Lu et al. (11) demonstrated that TBSA and argatroban use were significant predictors of plasma transfusions for burn patients. Similarly, another observational study reported that TBSA > 20% was a significant predictive factor for blood transfusion in burn adults and children (15). Remarkably, a retrospective study showed that transfused children with burn injuries had a mean TBSA of 29%–31% (16), a finding that corroborated with ours.

The study uncovered a significant association between WBC count and plasma transfusion, as previously reported in the literature. For example, Sen S et al. found that non-survivors had a higher WBC count than survivors during the first week after burn (17). Likewise, Niu L et al. reported a positive correlation between Injury Severity Score and WBC count (18). The present investigation similarly indicated that patients who received plasma transfusions had a notably higher WBC count compared to those who did not. This may be attributed to the significant fluid loss that often accompanies major burns, leading to alterations in blood profile. The combined effects of fluid loss and bone marrow response are known to cause leukocytosis, which is the most prevalent laboratory finding observed after a major burn 24 h post-injury (19).

Research findings suggested that heart rate can serve as a dependable indicator for the need of plasma transfusion in children with burns. A study conducted by Timothy H. Rainer et al. involving 1,891 major trauma patients provided evidence that heart rate levels of 120 /min and above were indicative of high risk for massive transfusion (20). Furthermore, a retrospective analysis of injured pediatric patients aged 1–18 years old from the Trauma Quality Improvement Project database, conducted by Travis M Sullivan et al., found that heart rate was a significant factor in determining the need for blood transfusion within 4 h of hospital arrival following injury (21). These studies highlighted the potential for heart rate to serve as a crucial predictive tool for plasma and blood transfusion requirements in children with burns and major trauma.

During the initial phase of burn shock, there is a remarkable increase in heart rate, followed by a subsequent reduction in arterial blood pressure. This change in heart rate serves as an early indicator for diagnosing burn shock. Following burn injury, body fluid loss and elevated release of vasoactive substances intensify myocardial contractility and heart rate, which in turn can function as a compensatory mechanism to increase cardiac output. However, a rapid heart rate also elevates myocardial oxygen consumption and reduces ventricular diastole, culminating in reduced myocardial contractility and diminished cardiac outputs.

Our study discovered that employing pre-hospital remedies upon arrival at the initial level of care significantly predicted a reduced likelihood of plasma transfusion being required in pediatric burn patients. This finding was in line with existing studies exploring the efficacy of formal pre-hospital treatments in burn cases. Pre-hospital interventions, such as using cold water to cool the burn area, safeguarding the wound, disinfecting the affected area, and expediting transportation to the nearest hospital, can positively impact clinical outcomes (22–24).

In this study, out of 117 (67.24%) pediatric burn patients who received plasma transfusion were not given pre-hospital remedies. This may be due to the fact that caregivers were expected to transfer the patients to the burn center quickly and hence bypassed any pre-hospital treatments. Moreover, only a small number of patients received cooling treatments that were sustained for less than 20 min. As a result, it is recommended that measures be taken to encourage the administration of pre-hospital treatments for pediatric burn patients, as outlined in previous studies (22, 25).

The transfusion rate of plasma in pediatric burn patients is influenced by various factors such as TBSA, WBC, heart rate, and pre-hospital remedies. These factors can help predict whether blood transfusions will be required during the treatment of major burns. Moreover, this also assists blood banks and healthcare providers in preparing for plasma transfusion.

## 5 Limitation

This study has several limitations that must be acknowledged. Firstly, it was a retrospective study, which only suggested apotential correlation and not a definitive causation. Secondly, this was a single-center retrospective study that included arelatively small sample size of pediatric burn patients. Thirdly, the plasma was transfused during the resuscitation period based on the severity of burns, it was not possible to match the plasma transfusion group and the non-plasma transfusion group according to the burn area and depth for prognosis comparison. Therefore, we did not conduct an analysis of the prognosis of plasma transfusion during the resuscitation period for children with burns.

## 6 Conclusion

The total body surface area, white blood cell, heart rate and pre-hospital remedies influenced the plasma transfusion rate in pediatric burn patients, which could predict whether blood transfusion would be needed during the treatment of major burns, and can further aid blood banks and clinicians in preparing for plasma.

## Data Availability

The original contributions presented in the study are included in the article/Supplementary Material, further inquiries can be directed to the corresponding author.
